# Metastatic pancreatic adenocarcinomas could be classified into M1a and M1b category by the number of metastatic organs

**DOI:** 10.1186/s12876-020-01431-8

**Published:** 2020-08-27

**Authors:** Fang Feng, Wei Cai, Gaoming Wang, Weigang Chen, Haochang Yang, Mingyu Sun, Li Zhou

**Affiliations:** 1 Department of Oncology, Suzhou Ninth People’s Hospital, Suzhou, 215200 China; 2 Department of Thoracic Surgery, Xuzhou Central Hospital, The Affiliated Xuzhou Hospital of Medical College of Southeast University, Xuzhou, 221009 China; 3 College of Clinical Medicine, Binzhou Medical University, Yantai, 264003 China; 4 Department of Breast Surgery, Xuzhou Central Hospital, The Affiliated Xuzhou Hospital of Medical College of Southeast University, Xuzhou, 221009 China

**Keywords:** Metastatic pancreatic adenocarcinoma, SEER, Overall survival, AJCC

## Abstract

**Background:**

With the improvement of treatment and prognosis for patients with late malignant diseases, certain malignancies with distant metastasis (M1 category) have been further classified into M1a (single metastatic site) and M1b (multiple metastatic sites) category in the staging system. We aimed to assess the feasibility of sub-classifying metastatic pancreatic adenocarcinoma (mPA) into M1a and M1b category depending on the number of metastatic organs.

**Methods:**

Patient records were collected from the Surveillance, Epidemiology, and End Results (SEER) database (2010–2015). Univariable and multivariable analyses were performed using the Cox regression model. Then survival analysis was determined using the Kaplan–Meier method.

**Results:**

A total of 11,885 patients were included in this analysis, including 9425 patients with single metastasis and 2460 patients with multiple metastases. Multivariable analysis showed that gender, age, marital status, grade, surgery, chemotherapy, and radiotherapy were independent prognostic factors for patients with single metastasis; gender, age, marital status, grade, chemotherapy and radiotherapy were independent prognostic factors for patients with multiple metastases. Notably, surgery was an independent prognostic factor for patients with single metastasis (*P* < 0.001) but not for patients with multiple metastases (*P* = 0.134). Kaplan–Meier analysis showed that patients with single metastasis (M1a) had better survival outcomes than patients with multiple metastases (M1b) (*P* < 0.001).

**Conclusions:**

PA patients with M1 diseases could be divided into M1a (single metastasis) category and M1b (multiple metastases) category by the number of metastatic organs. The subclassification would facilitate individualized treatment for late PA patients. Surgery was associated with lower mortality in M1a patients but not significantly in M1b patients.

## Background

Pancreatic cancer (PC), the fourth leading cause of cancer-related deaths in the United States, is a highly lethal and aggressive malignancy with a 5-year survival rate of less than 5% [1, 2]. Approximately half of PC patients have developed distant metastasis at the time of first diagnosis [3]. According to the current clinical guidelines for PC patients without distant metastasis, radical surgery together with chemotherapy is regarded as the primary treatment strategy although there are many borderline resectable and unresectable cases due to the location/local extension of the tumor. For metastatic PC patients (mPC), however, chemotherapy is recommended rather than surgery [4, 5]. Since accumulating evidence has demonstrated that primary tumor resection is beneficial to patients with metastatic malignancies [6–9], some researchers have explored whether local therapy has a favorable impact on mPC survival. Tao et al .[3] reported that surgical resection of primary tumors could prolong survival times in patients with mPC. Crippa et al. [10] found that surgical resection of primary pancreatic tumor was related to improved survival for mPC patients undergoing chemotherapy. These findings broadened the clinical perspective on surgical treatment for mPC.

With the improvement of treatment and prognosis for patients with late malignant diseases, certain malignancies with distant metastasis (M1 category) will be further classified into M1a (single metastatic site) and M1b (multiple metastatic sites) category in the staging system. For instance, M1 colorectal cancer (CRC) was further classified into M1a and M1b category in the 7th edition of American Joint Committee on Cancer (AJCC) staging manual [11]. M1 lung cancer was divided into M1a and M1b category in the International Association for the Study of Lung Cancer (IASLC) Staging Project [12]. It has not been investigated whether mPC patients should be further classified into subgroups yet. Hani et al. [13] reported that the number of metastatic organs was not significantly associated with the prognosis of mPC patients using the Surveillance Epidemiology and End Results (SEER) database. However, the result may be caused by their inappropriate inclusion criteria. The primary tumor of their study cohort should be restricted to “first sequence” rather than “two or larger sequence”. Some PA patients with a history of other malignancies would be included by the inappropriate inclusion criteria, which may lead to selection bias. Therefore, we aimed to assess the feasibility of sub-classifying metastatic pancreatic adenocarcinoma (mPA) into M1a and M1b category depending on the number of metastatic organs.

## Methods

### Patients

Data on patients with pancreatic adenocarcinomas were extracted from the SEER database, which is one of the largest clinical databases covering ~ 26% of the population in the United States [14]. Tumor site was coded as pancreas according to the International Classification of Diseases for Oncology (3rd edition, ICD–O–3). The further inclusion criteria were: (1) diagnosed between 2010 and 2015; (2) 18 years or older; (3) first primary tumor; (4) adenocarcinomas with positive histology; (5) distant metastasis with definite metastatic sites (liver, lung, bone and brain); (6) active follow-up information. Patients with distant metastasis (other organs rather than bone, liver, lung or brain) were excluded because they were not able to be assessed accurately based on the SEER data. Baseline patient characteristics included gender, age, race/ethnicity, tumor location, marital status, grade, tumor size, chemotherapy, radiotherapy, surgery and survival time. Follow-up time ranged from 0 to 70 months with a mean follow-up time of 5.7 months. The number of events for overall survival (OS) was 10,306 during the follow-up period.

### Statistical analysis

The factors associated with prognosis were determined by univariable and multivariable analyses using the Cox regression model and the proportional of hazards assumption was verified using Schoenfeld residuals. Kaplan–Meier analyses were used to evaluate the OS. All statistical analyses were performed using PASW Statistics 18. A two-sided *P*-value < 0.05 was considered statistically significant.

## Results

### Patient characteristics

A total of 11,885 patients with mPA met our inclusion criteria consisting of 9425 patients with single metastasis and 2460 patients with multiple metastases (Table 1). The entire cohort included 6564 male and 5321 female patients with a median age of 66 years (ranging from 19 to 100). Patients with tumors located in the body/tail of pancreas had a larger proportion (37.1%) than those in the head of pancreas (33.4%). Most patients were White (9362, 78.8%) and more than half (6634, 55.8%) were married. The proportion of patients with poorly differentiated or undifferentiated tumors was barely larger than those with well or moderately differentiated tumors. The majority of patients received chemotherapy (55.0%), followed by radiotherapy (5.2%) and surgery (4.6%).

**Table 1:** Clinopathologic characteristics of mPA patients

Variables	Total (***n*** = 11,885)	Single Metastasis (***n*** = 9425)	Multiple Metastases (***n*** = 2460)	***P*** value
**Gender**				0.089
Male	6564 (55.2%)	5168 (54.8%)	1396 (56.7%)	0.089
Female	5321 (44.8%)	4257 (45.2%)	1064 (43.3%)	
**Age (years)**				0.174
≤ 65	5725 (48.2%)	4570 (48.5%)	1155 (47.0%)	
> 65	6160 (51.8%)	4855 (51.5%)	1305 (53.0%)	
Range (median)	19–100 (66)	19–100 (66)	21–96 (66)	
**Race**				0.434
White	9362 (78.8%)	7427 (78.8%)	1935 (78.7%)	
Black	1622 (13.6%)	1297 (13.8%)	325 (13.2%)	
Others	901 (7.6%)	701 (7.4%)	200 (8.1%)	
**Tumor location**				< 0.001
Head	3972 (33.4%)	3371 (35.8%)	601 (24.4%)	
Body/tail	4409 (37.1%)	3405 (36.1%)	1004 (40.8%)	
Others	3504 (29.5%)	2649 (28.1%)	855 (34.8%)	
**Marital status**				0.549
Married	6634 (55.8%)	5274 (56.0%)	1360 (55.3%)	
Unmarried/unknown	5251 (44.2%)	4151 (44.0%)	1100 (44.7%)	
**Grade**				0.001
Well/moderately differentiated	1308 (11.0%)	1089 (11.5%)	219 (8.9%)	
Poorly differentiated/undifferentiated	1439 (12.1%)	1148 (12.2%)	291 (11.8%)	
Unknown	9138 (76.9%)	7188 (76.3%)	1950 (79.3%)	
Tumor size (mm)*				
Range (median)	0–950 (40)	0–900 (40)	0–950 (43)	
**Surgery**				< 0.001
No	11,335 (95.4%)	8948 (94.9%)	2387 (97.0%)	
Yes	550 (4.6%)	477 (5.1%)	73 (3.0%)	
**Chemotherapy**				0.013
No/unknown	5350 (45.0%)	4188 (44.4%)	1162 (47.2%)	
Yes	6535 (55.0%)	5237 (55.6%)	1298 (52.8%)	
**Radiotherapy**				< 0.001
No	11,270 (94.8%)	9061 (96.1%)	2209 (89.8%)	
Yes	615 (5.2%)	364 (3.9%)	251 (10.2%)	
**Metastatic sites**				< 0.001
Bone	1056	193	863	
Brain	111	21	90	
Liver	10,883	8523	2360	
lung	2678	688	1990	
* 2723 cases missing.				

### Factors associated with the prognosis

**Fig. 1:**
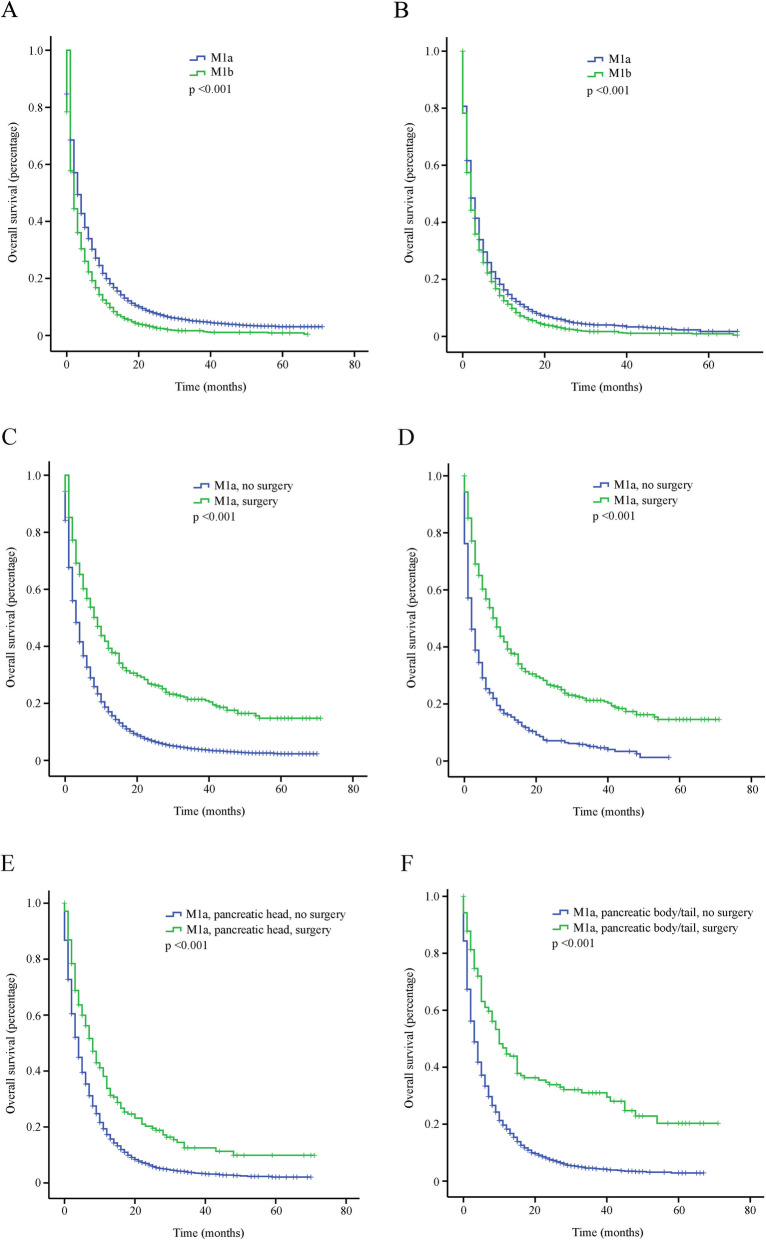
Graphs of overall survival (OS). **a** Entire cohort stratified by the number of metastatic organs: M1a (single metastatic site) and M1b (multiple metastatic sites). **b** Entire cohort after PSM. **c** M1a patients stratified by surgery. **d** M1a patients after PSM. **e** M1a patients with pancreatic head adenocarcinoma stratified by surgery. **f** M1a patients with pancreatic body/tail adenocarcinoma stratified by surgery

Using the Cox regression model, univariable and multivariable survival analyses were performed (Tables 2 and 3). Multivariable analysis showed that gender, age (≤65 yrs. vs. > 65 yrs), marital status (married vs. unmarried/unknown), grade (well/moderately differentiated vs. poorly differentiated/undifferentiated), surgery (no vs. yes), chemotherapy (no vs. yes) and radiotherapy (no vs. yes) were independent prognostic factors for patients with single metastasis; gender, age (≤65 yrs. vs. > 65 yrs), marital status (married vs. unmarried/unknown), grade (well/moderately differentiated vs. poorly differentiated/undifferentiated), chemotherapy (no vs. yes) and radiotherapy (no vs. yes) were independent prognostic factors for patients with multiple metastases. Notably, surgery was an independent prognostic factor for patients with single metastasis (*P* < 0.001) but not for patients with multiple metastases (*P* = 0.134). Kaplan–Meier analysis showed that patients with single metastasis (M1a) had better survival outcomes than those with multiple metastases (M1b) (*P* < 0.001, Fig. 1a). To reduce possible bias caused by potential confounding factors, a 1:1 propensity score matching (PSM) was performed. After PSM, 4874 patients (2437 patients each for M1a and M1b groups) were included. Similar results were observed (*P* < 0.001, Fig. 1b). Among M1a patients with PA, surgery predicted better survival outcomes (*P* < 0.001, Fig. 1c). After PSM, 950 patients (475 patients each for surgery and non- surgical groups) were included, and patients with surgery still had better survival outcomes (*P* < 0.001, Fig. 1d).

**Table 2:** Univariable analysis for mPA patients

Variables	Single Metastasis (***n*** = 9425)^**c**^		Multiple Metastases (***n*** = 2460)^**c**^	
	HR^**a**^(95% CI^**b**^)	***P*** value	HR (95% CI)	***P*** value
**Gender**				
Male	Reference		Reference	
Female	0.938 (0.898–0.980)	0.004	0.931 (0.856–1.013)	0.095
**Age (years)**				
≤ 65	Reference		Reference	
> 65	1.460 (1.397–1.526)	< 0.001	1.344 (1.235–1.461)	< 0.001
**Race**				
White	Reference		Reference	
Black	1.075 (1.009–1.145)	0.026	1.007 (0.890–1.140)	0.907
Others	0.981 (0.902–1.067)	0.655	1.053 (0.904–1.227)	0.505
**Tumor location**				
Head	Reference		Reference	
Body/tail	1.013 (0.962–1.067)	0.627	1.014 (0.912–1.128)	0.795
Others	1.138 (1.077–1.202)	< 0.001	1.093 (0.980–1.220)	0.112
**Marital status**				
Married	Reference		Reference	
Unmarried/unknown	1.231 (1.178–1.286)	< 0.001	1.246 (1.146–1.355)	< 0.001
**Grade**				
Well/moderately differentiated	Reference		Reference	
Poorly differentiated/undifferentiated	1.718 (1.566–1.884)	< 0.001	1.531 (1.273–1.840)	< 0.001
Unknown	1.630 (1.515–1.753)	< 0.001	1.411 (1.216–1.637)	< 0.001
**Tumor size**	1.000 (0.999–1.001)	0.767	1.000 (0.999–1.001)	0.426
**Surgery**				
No	Reference		Reference	
Yes	0.513 (0.461–0.572)	< 0.001	0.749 (0.584–0.961)	0.023
**Chemotherapy**				
No	Reference		Reference	
Yes	0.432 (0.413–0.452)	< 0.001	0.350 (0.320–0.382)	< 0.001
**Radiotherapy**				
No	Reference		Reference	
Yes	0.737 (0.657–0.827)	< 0.001	0.729 (0.635–0.838)	< 0.001

^a^*HR* hazard ratio

^b^*CI* confidence interval

^c^The number of events for patients with single metastasis and multiple metastases were 8077 and 2229, respectively

**Table 3:** Multivariable analysis for patients with metastatic pancreatic adenocarcinomas

Variables	Single Metastasis (***n*** = 9425)^**c**^		Multiple Metastases (***n*** = 2460)^**c**^	
	HR^**a**^ (95% CI^**b**^)	***P*** value	HR (95% CI)	***P*** value
**Gender**				
Male	Reference		Reference	
Female	0.889 (0.844–0.936)	< 0.001	0.869 (0.786–0.961)	0.006
**Age (years)**				
≤ 65	Reference		Reference	
> 65	1.333 (1.267–1.403)	< 0.001	1.259 (1.139–1.391)	< 0.001
**Race**				
White	Reference		Reference	
Black	1.058 (0.983–1.140)	0.132	0.984 (0.845–1.145)	0.834
Others	0.969 (0.879–1.069)	0.529	1.073 (0.895–1.286)	0.447
**Tumor location**				
Head	Reference		Reference	
Body/tail	1.053 (0.995–1.114)	0.073	1.041 (0.924–1.172)	0.510
Others	1.076 (1.005–1.152)	0.036	1.045 (0.911–1.199)	0.530
**Marital status**				
Married	Reference		Reference	
Unmarried/unknown	1.164 (1.104–1.226)	< 0.001	1.147 (1.036–1.270)	0.008
**Grade**				
Well/moderately differentiated	Reference		Reference	
Poorly differentiated/undifferentiated	1.952 (1.757–2.169)	< 0.001	1.522 (1.218–1.901)	< 0.001
Unknown	1.781 (1.637–1.937)	< 0.001	1.440 (1.201–1.726)	< 0.001
**Tumor size**	1.001 (1.000–1.002)	0.156	1.000 (0.998–1.001)	0.490
**Surgery**				
No	Reference		Reference	
Yes	0.460 (0.405–0.522)	< 0.001	0.773 (0.551–1.083)	0.134
**Chemotherapy**				
No	Reference		Reference	
Yes	0.428 (0.406–0.451)	< 0.001	0.360 (0.324–0.401)	< 0.001
**Radiotherapy**				
No	Reference		Reference	
Yes	0.819 (0.719–0.932)	0.003	0.799 (0.673–0.948)	0.010

^a^*HR* hazard ratio

^b^*CI* confidence interval

^c^The number of events for patients with single metastasis and multiple metastases were 8077 and 2229, respectively

Additionally, the effect of tumor location (head vs. body/tail) on the prognosis was further analyzed. Both patients with pancreatic head adenocarcinoma and those with pancreatic body/tail adenocarcinoma could benefit from surgery, and patients with pancreatic body/tail adenocarcinoma may benefit more (*P* < 0.001, Fig. 1e and f).

## Discussion

The prognosis of PA patients is rather poor and numerous attempts have been made to improve the present clinical situation. In the current study, we showed that PA patients with M1 diseases could be divided into M1a (single metastasis) category and M1b (multiple metastases) category by the number of metastatic organs. Surgery was an independent prognostic factor for M1a patients. However, similar but not significant survival benefit tendency was also observed in M1b patients with surgery. To our best knowledge, this is the first time to propose a subclassification of PA patients with distant metastasis. The subclassification would facilitate individualized treatment for late PA patients.

Chemotherapy was recommended for mPC patients in the clinical practice guidelines [15]. However, the slow development of new chemotherapeutic agents seriously restrained the improvement of long-term survival in mPC patients. During the past decades, most chemotherapy regimens have always been based on 5-fluorouracil and gemcitabine [16]. The emergence of molecule-targeted drugs did not even bring encouraging clinical benefits [17–19]. In this setting, more treatment options should be explored.

Surgery was considered as the best potential curative treatment for PC patients without distant metastasis but not indicated for mPC patients owing to its low safety and efficacy. With the remarkable progress of surgical techniques and procedures, extended surgical approaches can also be performed with low morbidity and mortality rates in well-selected mPC patients [20]. A SEER-based respective study showed that primary tumor resection could prolong the long-term survival of mPC patients [21]. However, they did not divided mPC patients into subgroups further according to metastatic sites. In our analysis, mPA patients with multiple metastases could not benefit from surgical treatment. In review of 23 mPC patients, Dünschede et al. [22] reported that surgery may improve survival for patients with metachronous liver metastases but not for those with synchronous liver metastases. Another study consisting of 22 cases also showed that simultaneous resection of primary tumor and liver metastases would not result in favorable survival outcomes [23]. These inconsistent results may be caused by small samples. More prospective and randomized clinical trials should be designed for mPC patients with M1a diseases to estimate the efficacy of surgery. Unfortunately, surgical treatment seemed not appropriate for mPC patients with M1b diseases, possibly owing to extremely high degree of malignancy and wide range of metastases.

There are some limitations in the present study that should be noted. First, all patient data are retrospective. Second, neoadjuvant chemotherapy and postoperative therapy were not distinguished in the SEER database. Neoadjuvant chemotherapy presents a potential opportunity for cure for patients with borderline resectable pancreatic cancer but remains rarely used for patients with metastatic pancreatic cancer. Presumably, less than 5% of entire cohort received neoadjuvant chemotherapy in our study since only 4.6% received surgical treatment. For patients with metastatic pancreatic cancer, systemic adjuvant therapies are the mainstay of treatment. Chemotherapy regimens were not provided either in the SEER database. The heterogeneity of chemotherapy regimens may create a bias in our findings. Third, there are no validation cohorts from other databases. Nevertheless, these findings provide novel insights for the clinical management of mPC patients.

## Conclusion

In sum, PA patients with M1 diseases could be divided into M1a (single metastasis) category and M1b (multiple metastases) category by the number of metastatic organs. The subclassification would facilitate individualized treatment for late PA patients. Surgery was associated with lower mortality in M1a patients but not significantly in M1b patients.

## Data Availability

The datasets generated and analyzed during the current study are available from the corresponding author on reasonable request.

## Abbreviations

PC: Pancreatic cancer
mPA: Metastatic pancreatic adenocarcinoma
SEER: Surveillance, Epidemiology, and End Results
CRC: Colorectal cancer
AJCC: American Joint Committee on Cancer
IASLC: International Association for the Study of Lung Cancer
PSM: Propensity score matching
OS: Overall survival
